# Case report about a successful full robotic radical gastric cancer surgery with intracorporeal robot-sewn anastomosis in a patient with situs inversus totalis and a two-and-a-half-year follow-up study

**DOI:** 10.1186/s12957-018-1311-z

**Published:** 2018-03-02

**Authors:** Hai-Bin Dai, Zhi-Chun Wang, Xiao-Bo Feng, Gang Wang, Wei-Yan Li, Chun-Hua Hang, Zhi-Wei Jiang

**Affiliations:** 1 Department of Anesthesiology, Jinling Hospital, School of Medicine, Nanjing University, No 305 East Zhongshan Road, Nanjing, 210002 China; 2 Department of Anesthesiology, Shuyang People’s Hospital, Jiangsu, China; 3 Department of General Surgery, Jinling Hospital, School of Medicine, Nanjing University, No 305 East Zhongshan Road, Nanjing, 210002 China; 4 Department of Neurosurgery, Drum Tower Hospital, School of Medicine, Nanjing University, No.321 Zhongshan Road, Nanjing, 210008 China

**Keywords:** Situs inversus, Gastric cancer, Minimally invasive surgery, Robot-assisted surgery, Gastrectomy

## Abstract

**Background:**

During the last decade, total laparoscopic and laparoscopic-assisted distal gastrectomy for gastric cancer patients has been developed as alternatives to open resection. In recent years, this minimally invasive surgery has been extended using robotic-assisted surgery.

**Case presentation:**

Here, we report a surgical intervention using a Da Vinci surgical robot in which a lower two-third stomach resection with subsequent Billroth II gastrojejunostomy was performed. The patient was a 53-year-old male with complete situs inversus gastric cancer who had received 2 cycles of neo-adjuvant oxaliplatin combined with S-1 medication. The operation took 3 h in total without complications. The amount of bleeding was about 50 mL, and on day 5 after the operation, the patient was discharged.

**Conclusions:**

This is the first report of a successful robot-assisted gastric cancer resection of advanced gastric cancer in a patient with the anatomical abnormality of situs inversus totalis.

## Background

Situs inversus, also called mirror-image malrotation, is a rare congenital abnormality defined by an anatomical variation in which the thoracic and abdominal organs are disposed in a mirrored position compared to the normal anatomy, including the heart, liver, spleen, and gallbladder. Its prevalence ranges from 1/8000 to 1/25,000 [1]. Most people with situs inversus totalis have no medical symptoms, but 25% of them develop primary ciliary dyskinesia, which causes chronic sinusitis and bronchiectasis. To date, six case reports of laparoscopic gastrectomy [2–6] and one case of partly robot-assisted gastrectomy [7] for situs inversus totalis patients have been published in English language journals, with most authors noting technical difficulties due to the unusual anatomy. In the present report, we describe a case in which a completely robot-assisted gastrectomy was performed on a patient with situs inversus totalis.

## Case presentation

**Fig. 1:**
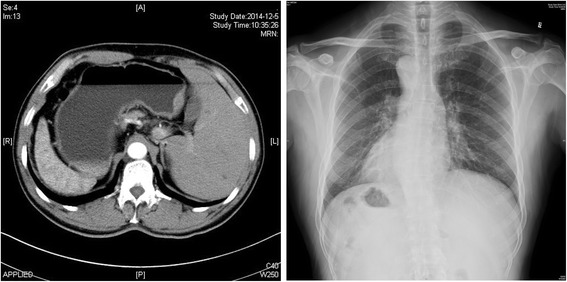
Images of preoperative abdominal CT and a chest X-ray film

**Fig. 2:**
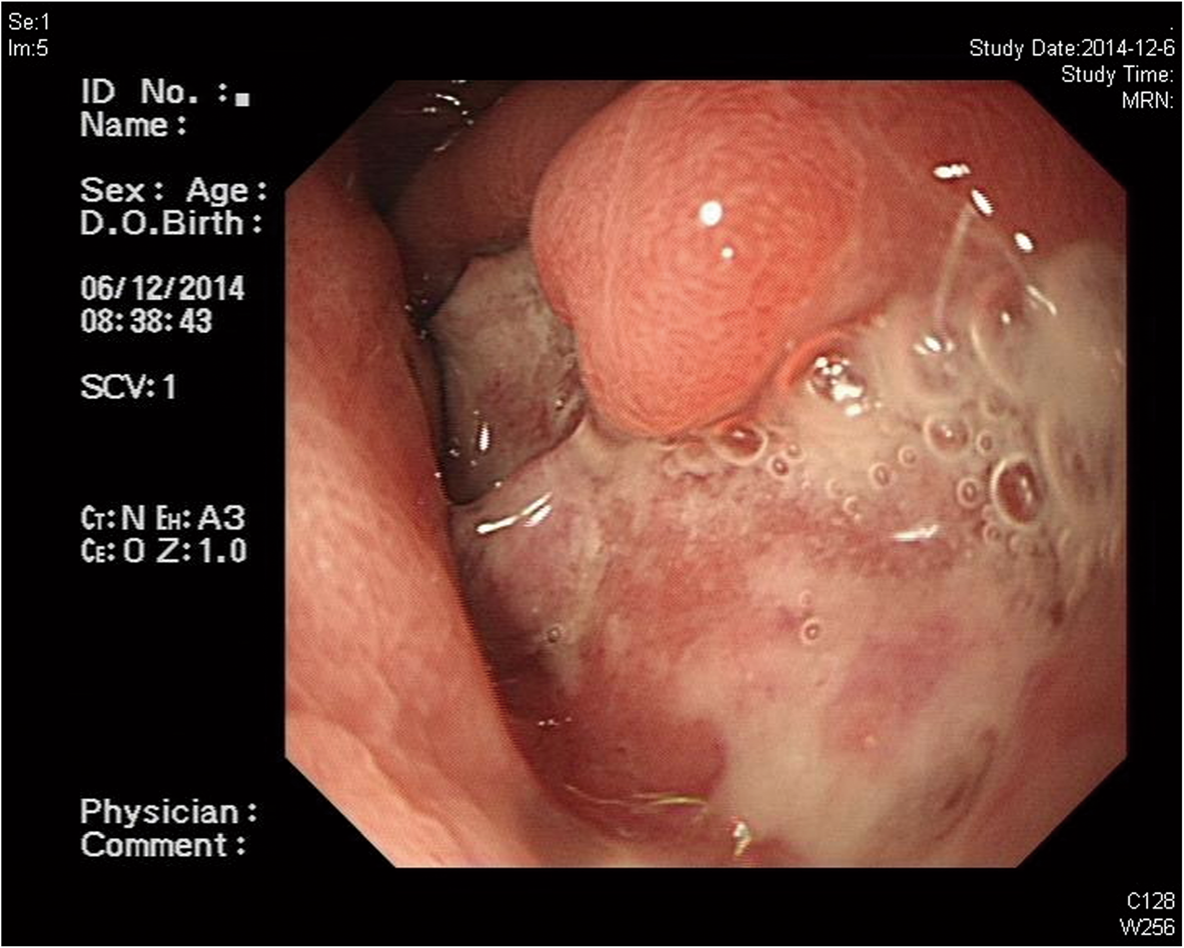
Results of the preoperative gastroscopy

The ethical committee of Jinling Hospital approved the study, and written informed consent was provided by the patient who was a 53-year-old male from Nantong City in China admitted to Jinling Hospital on Dec 5, 2014, because of epigastric bloating. On the admission day, contrast-enhanced computer tomography (CT) revealed distal gastric wall thickening and multiple enlarged lymph nodes. The visceral organs were rotated indicating situs inversus (Fig. 1). Upper endoscopy showed a nodulated hunch with irregular invaginations (6.0 cm × 6.0 cm) on the antrum of the stomach; the surrounding mucosa barrier was uplifted, leading to a diagnosis of gastric cancer (Borrmann type III) (Fig. 2). The biopsy results showed a moderately differentiated adenocarcinoma on the stomach antrum, which was C-erbB-2(2+).

Considering the tumor size and surrounding lymph node enlargements, two neo-adjuvant chemotherapy cycles of 0.2 g oxaliplatin i.v combined with 2 S-1 capsules (tegafur – gimeracil – oteracil potassium capsules) per day were administered, with each cycle lasting 3 weeks with a 7-day rest in between. The TNM classification has been T4aN2M0 before and T4aN1M0 after neo-adjuvant chemotherapy.

### Surgical technique

**Fig. 3:**
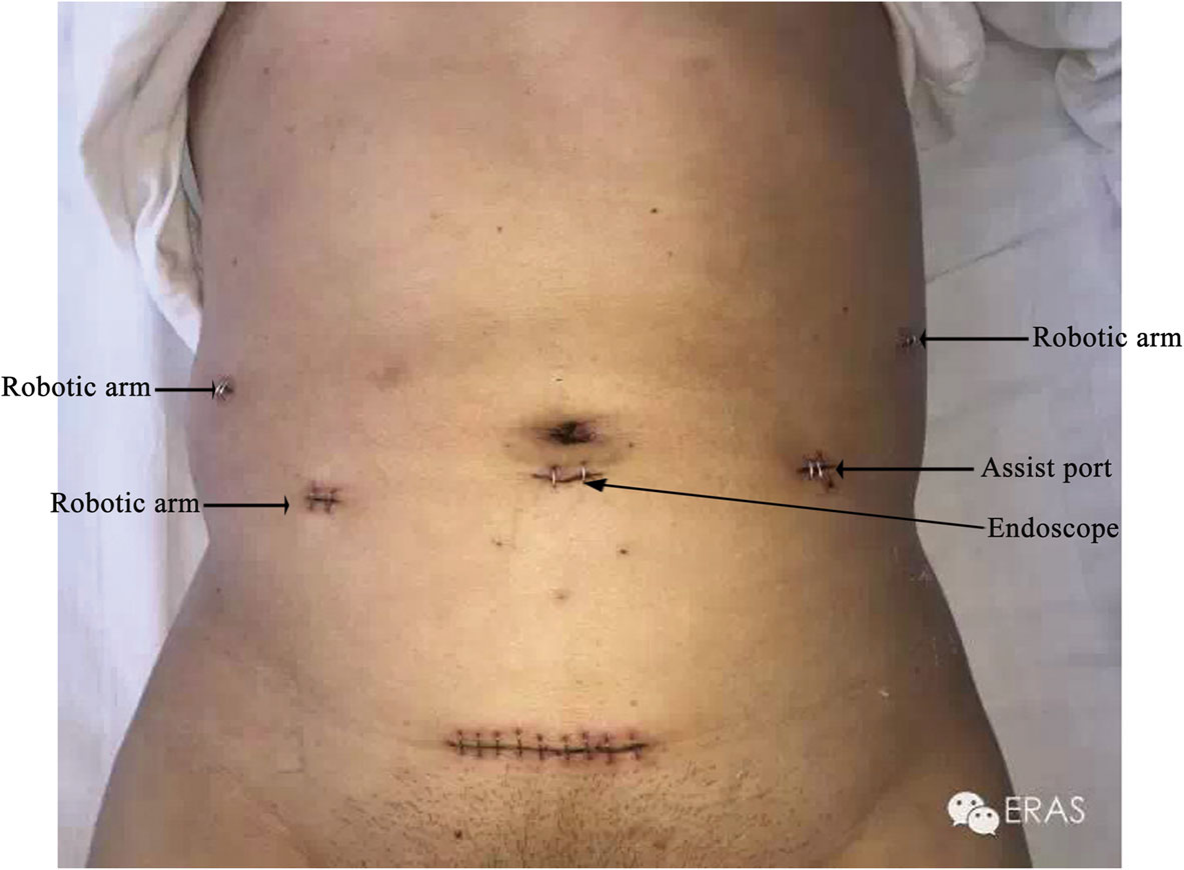
Locations of the abdominal incisions in the patient

On Feb 4, 2015, the patient underwent D2 gastrectomy under sevoflurane anesthesia. The operation was performed by an experienced surgeon, who had already carried out more than 700 robotic surgeries for gastric cancer. The surgical procedure was performed through five holes (Fig. 3).

**Fig. 4:**
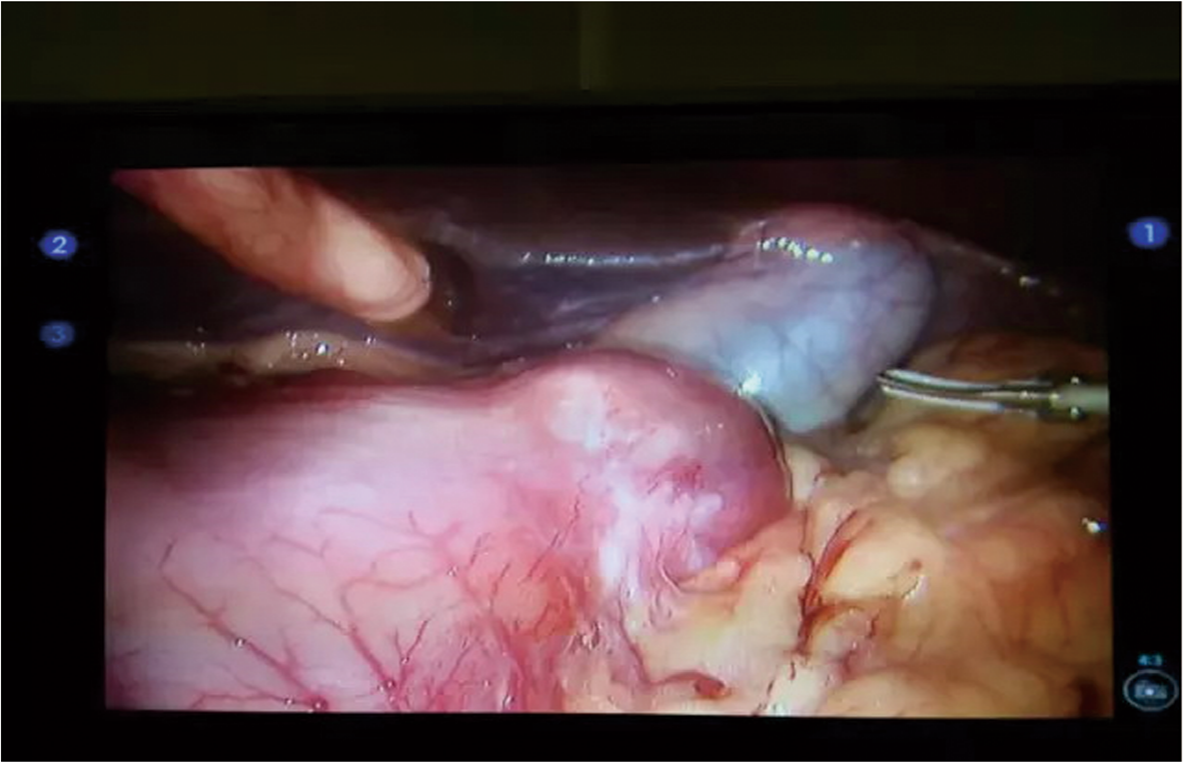
Intraoperative images of the liver and stomach disposed in a mirrored position (video screenshot from the robotic system)

First, a needle was placed in the umbilical hole, setting the pneumoperitoneum pressure to 12 mmHg with CO_2_. Then, four additional ports, 8 and 12 mm in diameter, were symmetrically inserted on both sides of the abdomen below the subcostal margin along midclavicular lines and in the anterior axillary line beside the umbilicus. The endoscope was inserted into the umbilical hole when the visceral organs in the mirrored positions could be observed (Fig. 4). No implantation metastasis was found in the abdomen.

Then, the mechanical arms of the robotic system were positioned in the abdominal cavity. A cutter, closure device, and ultrasonic knife as well as polymer ligating clips were used during the laparoscopic surgery. In the appropriate order, we freed and cut the ligaments around the stomach, then separated and clipped the corresponding arteries and veins, and finally removed the corresponding lymph node groups.

Facilitated by an endoscope and by using a 60-mm endoscopic stapler, first at about 2 cm distal to the pylorus, the duodenum was closed. Then, the lower two thirds of the stomach was cut off and the resected parts of the stomach, lymph nodes, ligaments, and omentum loaded inside a specimen bag in the abdominal cavity. Finally, a gastrojejunostomy was performed by the robotic system according to the Billroth II procedure in order to reconstruct the digestive tract, and a catheter was drawn from the left side of the abdominal wall into the abdominal cavity and fixed. Then, the robot device was removed.

Lastly, a transverse 5-cm incision was made in the hypogastric region (Fig. 3), through which the specimen bag was removed. The amount of bleeding was about 50 mL, and the operation lasted for a total of 3 h. The preoperative Hb value was 131 g/L, and the postoperative Hb value obtained the night after the operation and the next day was 119 and 118 g/L, respectively. The size of the tumor was about 2 cm × 3 cm and was located in the gastric antrum. Postoperative pathological reports showed poorly differentiated adenocarcinoma on the lesser curvature of the stomach and that the cancerous tissue had invaded the full wall. Metastasis was observed in the lymph nodes on the greater curvature (4/5), but not in the greater omentum and in the lymph nodes on the lesser curvature (0/15). On postoperative day 1, the patient left his bed and on day 2 after the operation, he had anal exhaust. On the third postoperative day, he drank a small amount of liquid and on day 5, he was discharged. One week after discharge, postoperative chemotherapy was initiated with the same prescription as preoperatively, which was continued for 4 cycles. Two and a half years after the operation, the patient did not develop a recurrence.

## Discussion and conclusions

The surgical treatment of disorders of the visceral organs in the abdominal cavity have evolved from traditional open surgery to laparoscopic surgery [8] and in recent years to the new minimally invasive techniques of robotic surgery [9]. The first published case of a situs inversus patient with gastric cancer, who had been treated with laparoscopy-assisted gastrectomy was in 2003 [2]. Later, other case reports followed, [3–6, 10] with most surgeons experiencing difficulties due to the abnormal position of the stomach. For example, Futawatari et al. [10] noted that during surgery, the surgical field was difficult to see, and thus, confirmation of anatomy was impeded. Therefore, two monitors were used to show the left and right sides, which were shifted throughout the operation [10]. Seo et al. reported that the first assistant had difficulties in the orientation of the entire anatomy, and the operators’ hand was confused [5]; Min et al. noted that the first assistant is expected to do more, as some of the structures that are easily approached in normal patients are more conveniently accessed by the first assistant in patients with situs inversus totalis [4]. In 2012, Kim et al. published a case report of a partly robot-assisted gastrectomy of a situs inversus patient, but the Billroth II gastrojejunostomy procedure was carried out extra corporally [7]. There are only few case reports about situs inversus gastric cancer patient treatments in China, but all of the patients received open surgery [11–13].

In gastric cancer surgery for mirror-image patients, a common problem is that the surgeon has to stand close to one side of the patient. The position has to be changed compared to the routine position adopted during the procedure, no matter if the intervention is open or laparoscopic surgery. However, when performing robotic surgery, neither the robotic system nor the surgeon’s position needs to be changed. The robotic system remains on the left side of the patient’s head for both normal and mirror-image patients. Moreover, the mechanical arms in the abdominal cavity are much more flexible with more dimensions of activity, and the sewing process is particularly fast compared with human hands. The dimensions of activity make it easier to remove lymph nodes located in the deep side of the abdomen or behind the viscera [14]. A minor drawback of the robotic maneuver is the lack of tactile or haptic feedback during the procedure. In our hospital, robotic surgery for patients with gastric cancer usually takes about 2.5 h, with the mirror-image patient only taking another 30 min. Although our patient was weakened by a neo-adjuvant chemotherapy cycle, he was well enough to leave the hospital 5 days after the minimally invasive intervention.

In conclusion, a minimally invasive surgery completely performed with a Da Vinci surgical robot is a feasible, safe, and accurate method to treat a situs inversus totalis advanced gastric cancer patient who had previously received neo-adjuvant chemotherapy.

## Abbreviation

CT: Computer tomography
